# Whole-Genome Analysis of *Salmonella enterica* Serovar Enteritidis Isolates in Outbreak Linked to Online Food Delivery, Shenzhen, China, 2018

**DOI:** 10.3201/eid2604.191446

**Published:** 2020-04

**Authors:** Min Jiang, Feng Zhu, Chao Yang, Yinhua Deng, Patrick S.L. Kwan, Yinghui Li, Yiman Lin, Yaqun Qiu, Xiaolu Shi, Hui Chen, Yujun Cui, Qinghua Hu

**Affiliations:** Shenzhen Center for Disease Control and Prevention, Shenzhen, China (M. Jiang, C. Yang, P.S.L. Kwan, Y. Li, Y. Lin, Y. Qiu, X. Shi, Q. Hu);; Nanshan District Center for Disease Control and Prevention, Shenzhen (F. Zhu, H. Chen);; State Key Laboratory of Pathogen and Biosecurity, Beijing Institute of Microbiology and Epidemiology, Beijing, China (C. Yang, Y. Cui);; College of Life Science, Sichuan University, Sichuan, China (Y. Deng)

**Keywords:** Salmonella enterica serovar Enteritidis, outbreak, foodborne illness, epidemiology, whole-genome sequencing, online food delivery, bacteria, China, acute gastroenteritis, food safety

## Abstract

In July 2018, an outbreak of 10 cases of *Salmonella enterica* serovar Enteritidis infection occurred in Shenzhen, China. Outbreak investigation complemented by whole-genome sequencing traced the source to food ordered online. Our investigation highlights the role of online food delivery platforms as a new mode of foodborne disease transmission.

*Salmonella* spp. are a leading bacterial cause of acute gastroenteritis globally, resulting in ≈93.8 million cases of gastroenteritis and ≈155,000 deaths each year (*1*). *Salmonella enterica* serovar Enteritidis has been the most common cause of *Salmonella* infections, accounting for >40% of human cases worldwide and >30% in China (*2*,*3*). This serovar frequently has been isolated in foodborne disease outbreaks globally, which are often associated with poultry and related products, such as shell eggs (*4*,*5*).

In China, the use of online food delivery services has gained substantial popularity with the advent of smartphone mobile applications and the development of online food delivery platforms. The number of online food delivery orders has increased rapidly in recent years, accounting for ≈10 billon orders and >100 million monthly active users in 2018 (*6*). However, while such services are convenient, the complex spatiotemporal dynamics of online food delivery networks could result in a new means of spreading foodborne diseases and pose previously unknown effects on public health. We investigated an outbreak complemented by the use of whole-genome sequencing (WGS) to identify and delineate outbreak and sporadic cases to confirm the source of a *Salmonella* Enteritidis outbreak linked to food ordered through an online food delivery platform in Shenzhen, China.

## The Study

During June 30–July 3, 2018, a total of 10 cases of diarrheal disease were reported at 2 hospitals in the Nanshan District of Shenzhen, China. This outbreak was suspected to be foodborne illness and was notified to the Shenzhen Center for Disease Control and Prevention (Shenzhen CDC). We collected details of food exposure histories, clinical manifestations, and demographic data through interviews with ill persons.

A total of 21 samples were collected during laboratory and environmental investigations, comprising anal swab specimens from 7 case-patients and 14 samples from the implicated restaurant (6 from chicken legs, 4 from restaurant staff, 2 from kitchenware items, and 2 from other foods). All samples were forwarded to the laboratory-based testing of common foodborne pathogens at Shenzhen CDC as previously described (*7*). *Salmonella* Enteritidis isolates were subtyped by pulsed-field gel electrophoresis (PFGE) using XbaI according to standard PulseNet protocols (https://www.cdc.gov/pulsenet/pathogens/protocols.html).

We sequenced genomes on the BGISEQ-500 platform (MGI Tech Co., Ltd., https://en.mgitech.cn) to generate 100-bp paired-end reads and deposited short-read sequence data in GenBank under BioProject PRJNA565566. We used Snippy version 4.3.8 (https://github.com/tseemann/snippy) for single-nucleotide polymorphism (SNP) calling, with *Salmonella* Enteritidis P125109 (GenBank accession no. NC_011294) as the reference genome. We excluded SNPs located in repetitive and recombinogenic regions before phylogenetic analysis, identified repetitive regions using TRF version 4 (https://tandem.bu.edu/trf/trf.html) and self-aligning by blastn (https://blast.ncbi.nlm.nih.gov), and determined recombinogenic regions by using Gubbins version 2.3.4 (*8*). We constructed a maximum-likelihood tree based on genomewide SNPs from an alignment of 300 SNPs using RAxML version 8.2.12 (*9*) under the general time-reversible with gamma distribution model (100 bootstraps).

All 10 case-patients were university students who had diarrhea (>3×/24 h), fever (>37.5°C), and high leukocyte counts (>10.0 × 10^9^ cells/L [reference 4.0–10.0 × 10^9^ cells/L]); 7 case-patients also reported nausea and vomiting. Case-patients were from 6 different colleges of the same university but lived in different dormitories and did not know each other. However, on the afternoon of June 30, all had eaten food delivery (chicken leg with rice) from the same restaurant near the university, ordered through an online delivery platform during a 6-hour period (noon–6 pm). Foods were precooked an hour before anticipated orders and left at room temperature and then dispatched upon receipt of orders and delivered within 1 hour in ambient temperature (29°C) using a linen storage bag.

From a total of 21 samples, 9 were positive for *Salmonella* Enteritidis, which was isolated from 5 chicken legs and from 4 case-patients and belonged to an indistinguishable XbaI PFGE pattern (JEGX01.SZ0001). Interrogation of existing PFGE patterns within the Shenzhen CDC PulseNet local database showed that 5 *Salmonella* Enteritidis isolates from sporadic cases within 1 month before the outbreak shared the same PFGE pattern. Routine surveillance further identified 5 additional sporadic isolates with the same PFGE pattern within 1 month after the outbreak. However, no clear epidemiologic links were found between any of the 10 sporadic cases and the outbreak.

WGS SNP-based cluster analysis showed that all 9 outbreak-associated isolates were genetically closely related to each other (Figure). Isolates from 5 chicken legs and 4 case-patients differed by <1 SNP, confirming chicken legs as the food source of the outbreak. In comparison, the minimum distance between the 10 sporadic isolates and any of the outbreak-associated isolates was 59 SNPs, larger than the common threshold (<3 SNPs) used for delineating outbreak clusters (*10*), indicating that the sporadic cases were not part of the outbreak.

**Figure:**
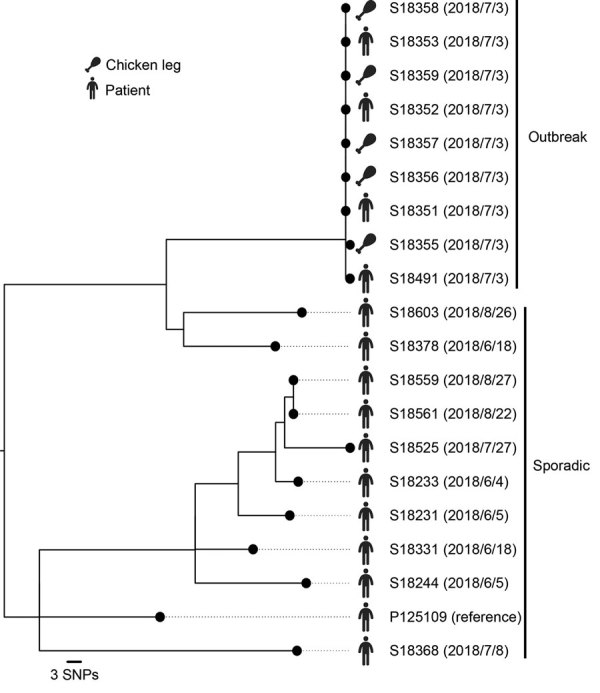
Clustering of 19 outbreak and sporadic *Salmonella enterica* serovar Enteritidis isolates from an outbreak linked to online food delivery, Shenzhen, China, 2018. Clusters were inferred by constructing a maximum-likelihood tree based on 300 genomewide SNPs. Isolation dates are provided in parentheses adjacent to isolate numbers; reference genome P125109 was used for SNP calling. Scale bar indicates nucleotide substitutions per site. SNPs, single-nucleotide polymorphisms.

## Conclusions

The burgeoning online food delivery industry has resulted in a landscape of change in food consumption behavior and lifestyle in China. In contrast to traditional restaurant dining, online food delivery could send potentially contaminated food across wide geographic areas throughout a city within a short time to cause large-scale outbreaks. Online food delivery also poses additional food safety risks, including improper handling and storage temperature during transport. As illustrated in this outbreak, the total time elapsed was 2 hours from food preparation to delivery at ambient temperature, potentially enabling *Salmonella* Enteritidis to sufficiently multiply and cause illnesses.

On the basis of the fine-scale delineation by whole-genome SNP-based cluster analysis, we differentiated genetically closely related *Salmonella* Enteritidis isolates that were indistinguishable by PFGE. WGS confirmed chicken legs as the outbreak source and excluded the possible links of additional sporadic isolates to the outbreak and highlights the advantages of using WGS for differentiating highly clonal *Salmonella* Enteritidis isolates. Several studies have demonstrated high epidemiologic concordance and superior discriminatory power of WGS in retrospective outbreak analyses involving *Salmonella* Enteritidis (*10*,*11*).

In this report of a *Salmonella* Enteritidis outbreak investigation linked to online food delivery guided by WGS, we highlighted the food safety challenges posed by a new mode of foodborne disease transmission. One of the new features of this outbreak is that the case-patients who ate food from the same source were isolated from each other, which contrasts with typical foodborne outbreaks that usually involved dining in restaurants or catering events within a family or group settings. Additional cases might therefore be more likely to be missed during the epidemiologic investigation. However, detailed information associated with food orders, such as the ordering and delivery time, the food items ordered, and the names and addresses of the merchant and the consumer, would all be electronically recorded over the online food delivery platform. Therefore, such remarkable level of detail would be highly valuable for prospective outbreak investigations. Close collaboration between public health agencies and online food delivery platforms would be essential to facilitate timely intervention of disease propagation and limit the scale of outbreaks efficiently.

Given the continued rapid growth anticipated for the online food delivery industry, in-depth risk assessments should be a research priority to inform appropriate food safety strategies. We explored a rare opportunity to investigate and gained new insights into the transmission dynamics of a *Salmonella* Enteritidis outbreak over an Internet platform, complemented with the pragmatic use of WGS analysis.

## References

[1] Majowicz SE, Musto J, Scallan E, Angulo FJ, Kirk M, O’Brien SJ, et al.; International Collaboration on Enteric Disease ‘Burden of Illness’ Studies. The global burden of nontyphoidal Salmonella gastroenteritis. Clin Infect Dis. 2010;50:882–9. 10.1086/650733 20158401

[2] Hendriksen RS, Vieira AR, Karlsmose S, Lo Fo Wong DM, Jensen AB, Wegener HC, et al. Global monitoring of *Salmonella* serovar distribution from the World Health Organization Global Foodborne Infections Network Country Data Bank: results of quality assured laboratories from 2001 to 2007. Foodborne Pathog Dis. 2011;8:887–900. 10.1089/fpd.2010.0787 21492021

[3] Ran L, Wu S, Gao Y, Zhang X, Feng Z, Wang Z, et al. Laboratory-based surveillance of nontyphoidal *Salmonella* infections in China. Foodborne Pathog Dis. 2011;8:921–7. 10.1089/fpd.2010.0827 21492026

[4] Pijnacker R, Dallman TJ, Tijsma ASL, Hawkins G, Larkin L, Kotila SM, et al.; International Outbreak Investigation Team. An international outbreak of *Salmonella enterica* serotype Enteritidis linked to eggs from Poland: a microbiological and epidemiological study. Lancet Infect Dis. 2019;19:778–86. 10.1016/S1473-3099(19)30047-7 31133519

[5] Centers for Disease Control and Prevention. Surveillance for foodborne disease outbreaks United States, 2017: annual report. Atlanta: The Centers; 2017.

[6] Yang X. Annual analysis of internet takeaway market, China, 2018 [cited 2019 Apr 9]. https://www.analysys.cn/article/analysis/detail/20019271

[7] Hu Q, Coburn B, Deng W, Li Y, Shi X, Lan Q, et al. *Salmonella enterica* serovar Senftenberg human clinical isolates lacking SPI-1. J Clin Microbiol. 2008;46:1330–6. 10.1128/JCM.01255-07 18272702 PMC2292908

[8] Croucher NJ, Page AJ, Connor TR, Delaney AJ, Keane JA, Bentley SD, et al. Rapid phylogenetic analysis of large samples of recombinant bacterial whole genome sequences using Gubbins. Nucleic Acids Res. 2015;43:e15. 10.1093/nar/gku1196 25414349 PMC4330336

[9] Stamatakis A. RAxML version 8: a tool for phylogenetic analysis and post-analysis of large phylogenies. Bioinformatics. 2014;30:1312–3. 10.1093/bioinformatics/btu033 24451623 PMC3998144

[10] Taylor AJ, Lappi V, Wolfgang WJ, Lapierre P, Palumbo MJ, Medus C, et al. Characterization of foodborne outbreaks of *Salmonella enterica* serovar Enteritidis with whole-genome sequencing single nucleotide polymorphism-based analysis for surveillance and outbreak detection. J Clin Microbiol. 2015;53:3334–40. 10.1128/JCM.01280-15 26269623 PMC4572550

[11] Deng X, Shariat N, Driebe EM, Roe CC, Tolar B, Trees E, et al. Comparative analysis of subtyping methods against a whole-genome-sequencing standard for *Salmonella enterica* serotype Enteritidis. J Clin Microbiol. 2015;53:212–8. 10.1128/JCM.02332-14 25378576 PMC4290925

